# Monkeypox: New epidemic or fake news? Study of psychological and social factors associated with fake news attitudes of monkeypox in Italy

**DOI:** 10.3389/fpsyg.2023.1093763

**Published:** 2023-02-17

**Authors:** Filippo Maria Nimbi, Guido Giovanardi, Roberto Baiocco, Annalisa Tanzilli, Vittorio Lingiardi

**Affiliations:** ^1^Department of Dynamic and Clinical Psychology and Health Studies, Sapienza University of Rome, Rome, Italy; ^2^Department of Social and Developmental Psychology, Sapienza University of Rome, Rome, Italy

**Keywords:** monkeypox, fake news, epistemic trust, mentalized affectivity, health, COVID-19, hoax, credulity

## Abstract

**Objective:**

Starting from May 2022, a growing number of monkeypox cases have been identified in several countries in Europe and the United States. To date, information on social reaction to the news circulating about monkeypox is limited. Assessing psychological and social elements related to the tendency to misinterpret monkeypox information is urgent and useful in setting up tailored education and prevention programs for specific populations. The present study aims to explore the association of selected psychological and social variables to monkeypox attitudes as fake news.

**Methods:**

Three hundred and thirty-three participants (212 women, 110 men, and 11 other genders) from the general Italian population completed nine self-report measures.

**Results:**

Results showed that people that were more likely to believe that monkeypox was a hoax were: older, heterosexual, politically conservative, and more religious. Moreoverm they were more likely to show more negative attitudes toward gay men, higher levels of sexual moralism, less knowledge and fear about monkeypox, no previous infections of COVID-19, lower number of COVID-19 vaccine doses, and being closer to no-vax theories. On the psychological side, participants that were more likely to believe that the monkeypox was a hoax were associated with lower levels of epistemic trust and order traits, with higher levels of epistemic mistrust, close-mindedness, and ability to process emotions. A full mediation model which explores the relationships between the main variables related to fake news attitudes toward monkeypox was tested, reporting good fit indices.

**Conclusion:**

Results from the current study could be helpful to improve the effectiveness of health communication, design targeted education, and support people to engage in healthier behaviors.

## Introduction

Monkeypox is a zoonotic infection caused by a virus in the same family as smallpox (Poxviridae), which was first identified in 1958 in African monkey colonies (Magnus et al., 1959) and reported on humans in 1970 (Bunge et al., 2022). Starting from 18 May 2022 (ECDC-European Center for Disease Prevention and Control, 2022a), a growing number of monkeypox cases has been identified in several countries in Europe and United States. Monkeypox has been shown to be transmitted through close respiratory droplets and physical interaction (skin-to-skin), especially during prolonged and intimate contact, such as in sexual activity. It can also spread through sharing contaminated objects, such as clothes, bedding, and sex toys (ECDC-European Center for Disease Prevention and Control, 2022b). A recent metanalysis (Bragazzi et al., 2022) underlined that the ongoing breakout seems to differ from previous monkeypox epidemics in terms of age (54.29% of individuals are around 30 vs. 4–21 years old in previous outbreaks), sex/gender (most cases being cisgender men vs. no gender difference in previous outbreaks), and way of transmission (sexual contact vs. droplets or contamination). Moreover, the clinical presentation seems atypically characterized by anogenital lesions and rashes reported in about 1/3 of the cases, in addition to typical symptoms such as chills, exhaustion, fever, headache, muscle aches and backache, and swollen lymph nodes (Bragazzi et al., 2022).

Public health services are currently facing the new Monkeypox outbreak and taking measures to counter its diffusion (ECDC-European Center for Disease Prevention and Control, 2022b). Therefore, health communication has a key role at this stage, to increase prevention and community engagement strategies (Schmidt-Sane et al., 2022; WHO–World Health Organization, 2022). The risk of stigmatizing populations such as men who have sex with men (MSM) and African people is high, so much that the “Social Science in Humanitarian Action (SSHAP)” has produced a document (Schmidt-Sane et al., 2022) suggesting how to better communicate about this emergency. A leading message is to prevent stigma and misconceptions, such as having campaings targeted to more at risk groups. In addition to this, it is important to inform other groups that may be not seen as exposed, addressing possible stigmatizing behaviors toward vulnerable groups. Health policies should consider what has been learned from past epidemics like HIV/AIDS and COVID-19. In both cases, a dissemination of fake information has fueled fear and discrimination toward specific populations (MSM, transgender people, sex workers, people who inject drugs, and Chinese people’ as not exclusive to such populations), perpetrating stigma that persists in the current days (Starks et al., 2013; Hedge et al., 2021; Schmidt-Sane et al., 2022). Moreover, there is evidence of the role of misinformation, disinformation, and related ideologies (anti-mask, anti-vaccine, and anti-5G) related to health communication (Das and Ahmed, 2022).

Health misinformation may exacerbate infectious disease outbreaks; especially the unconditional circulation of ruinous advice such as “fake news” and “hoaxes,” which are constructed with no respect for accuracy and frequently integrated with emotive or conspiracy-framed narratives (Brainard and Hunter, 2020). To date, information on social reactions to the news circulating about monkeypox is limited and referring mainly to previous monkeypox outbreaks in sub-Saharan countries (Okanume, 2018; Nnaemeka and Onunkwor, 2021). A preliminary study on Twitter’s most popular reactions to the monkeypox May 2022 outbreak (Ortiz-Martínez et al., 2022a) showed that most tweets were posted by informal individuals or groups (60%), followed by healthcare/public health (32%) and news outlets or journalists (8%). Regarding the topic, the majority (52%) included misinformation and unverifiable information, 20% humorous/non-serious content, and only 28% provided significant information regarding the monkeypox. The misleading tweets were more likely to receive replies, retweets, and likes than the medically correct content. These early results show how confusing information can be received and disseminated by the public, generating a, problematic environment in which to circulate accurate and socially useful health information. A study on YouTube monkeypox videos carried out at the end of May 2022 (Ortiz-Martínez et al., 2022b) showed similar results, with misleading videos having a higher number of likes and views when compared to useful/scientifically accurate videos. The majority presented messages related to conspiracy theories about fake outbreaks created by international companies to sell vaccines and monkeypox as a disease only transmitted through sexual intercourse and exclusively related to MSM.

Reducing harmful circulating information, making the population unable to share or believe dangerous advice, and mitigating the influence of bad information in disease outbreak outcomes may represent health priorities in facing epidemics (Brainard and Hunter, 2020; Moscadelli et al., 2020). Some psychological aspects (personality traits, cognitive closure) and social attitudes (attitudes toward sexuality, COVID-19 pandemic) can play a key role in explaining how health-related information is interpreted (salience for the person) and can direct health behavior (such as seeking clinical assessment or preventive vaccination). Exploring the role of these factors can help to create more appropriate and effective communication, capable of reaching a wider group of people. In this sense, research in this field is urgent and useful to set up tailored education and prevention programs and actions for specific populations (Pakpour and Griffiths, 2020; Schmidt-Sane et al., 2022). It would be beneficial to study attitudes to monkeypox as a hoax/fake news and the possible role of some sociodemographic variables, such as gender, age, education, political and religious attitudes, as well as attitudes related to the COVID-19 pandemic management (which brought to the forefront the issue pertaining to fake news and conspiracy theories; van Der Linden et al., 2020). Furthermore, in line with the early data that associated the monkeypox epidemic with the MSM population (Bragazzi et al., 2022; ECDC-European Center for Disease Prevention and Control, 2022a,b), an exploration of the role of attitudes toward LGBQI+ population and sexual moralism might also be of interest to deepen this issue.

Other relevant concept are the epistemic trust, mentalized affectivity, and the need for cognitive-closure. The first one (Fonagy and Allison, 2014) is referred to the individual’s willingness to regard new knowledge as relevant and reliable and therefore worthy of integration into the personal life. In contrast, epistemic distrust is characterized by inflexible patterns of thinking and the difficulty of learning from the social environment. Mentalized affectivity (Jurist, 2005) is defined as the cognitive and affective ability to understand our own and others’ thoughts and feelings. The affective regulation of emotion is based on the capacity for mentalization. In this sense, emotions are not only controlled in the process of regulation, but are also reevaluated in meaning in a process that involves identifying, processing, and expressing the emotion. Theoretically, we can observe a significant overlap of mentalized affectivity with other psychological frameworks, such as theory of mind, emotional regulation and emotional intelligence. Especially the latter has been seen as having a relationship with the ability to recognize or not fake news shared in social media (Preston et al., 2021). The need for cognitive closure may be described as the desire to have an answer on a given topic, regardless of the type of response, rather than remain in ambiguity or confusion (Roets and Van Hiel, 2011). This trait is described along a continuum from a high need for closure at one end and an increased need to avoid closure at the other end. We expect that all these factors may play a role in determining distrust attitudes concerning the information circulating about monkeypox in the 2022 outbreak. In this sense, the analysis of psychological constructs which are closely connected to information reliability and emotional regulation, such as epistemic trust, mentalized affectivity, and the need for cognitive closure, may be helpful for researchers and policymakers.

## Aims

Studies on people’s emotional and psychological reaction to the monkeypox epidemic gripping Europe and Western countries are lacking since the outbreak is highly recent (May 2022). This study investigates possible predictors of fake news attitudes to monkeypox information chosen from a set of psychological and social variables of interest based on the literature on previous epidemics such as COVID-19 and HIV.

Specifically, the first objective is to investigate the effect of (1) sociodemographic variables, (2) attitudes and knowledge toward monkeypox separately and lived to experience concerning the COVID-19 pandemic, (3) psychological variables such as epistemic trust, need for closure, psychopathological traits, mentalized affectivity, and (4) attitudes toward the LGBT+ (Lesbian, Gay, Bisexual, Transgender, and other identities) community and sexuality. The second objective aims to identify the best predictors of fake news attitudes and test a path diagram that explores the relationships between the main variables related to fake news attitudes toward monkeypox, discussing some possible implications for health and prevention policies.

## Methods

### Participants and procedures

Three hundred forty-two volunteers (218 women, 112 men, and 12 people of other genders) from the Italian general population participated in the current study. People were recruited using a snowball technique, sharing sponsored advertisements on institutional websites, and social networks (e.g., Facebook, Instagram, and LinkedIn). The web survey was available on Google.forms platform and answers were collected in June 2022. Participants completed an informed consent form before accessing the survey. They had access to participation information sheet with opportunity to ask questions about participation in advance of giving consent by mail. The questionnaire administered was anonymous, and no remuneration was provided to participants. The institutional ethics committee of the Department of Dynamic and Clinical Psychology and Health Studies, Sapienza University of Rome, approved this study on June 13, 2022.

Inclusion criteria were being aged 18 or over and being fluent in Italian. Nine responses (2.63%) were excluded from the present study because they represented duplicated, falsified, or incomplete records. The final group resulted in 333 participants (212 women, 110 men, and 11 other genders). Sociodemographic data of the participants are presented in Table 1. The same dataset was used for a preliminary study on predictors of monkeypox fear (under review).

**Table 1:** Sociodemographic variables description.

Variables		Participants (*n* = 333)
		M ± ds (min-max)
Age		31.71 ± 11.14 (18–71) Q_3_– Q_1_: 22–40
		*n* (%)
Sex assigned at birth	Female	217 (65.17)
	Male	116 (34.83)
Gender	Female	212 (63.66)
	Male	110 (33.03)
	Transgender	1 (0.3)
	Non-binary spectrum	4 (1.2)
	Currently exploring gender identity	6 (1.8)
Sexual orientation	Heterosexual	237 (71.17)
	Bisexual	31 (9.31)
	Lesbian/Gay	50 (15.02)
	Pansexual	11 (3.3)
	Asexual	4 (1.2)
Marital status	Unmarried	269 (80.78)
	Married	53 (15.92)
	Separated	10 (3)
	Widowed	1 (0.3)
Relational status	Single	162 (48.65)
	Couple	160 (48.05)
	Polyamory	11 (3.3)
Education level	Middle school	16 (4.8)
	High School	140 (42.04)
	University	131 (39.34)
	PhD and postgrads courses	46 (13.81)
Work status	Unemployed	32 (9.61)
	Employed	179 (53.75)
	Student	120 (36.04)
	Retired	2 (0.6)

### Measures

For this study, nine self-report measures assessing different psychosocial variables were administered for about 25 min (24.86 ± 6.78).

#### Sociodemographic questionnaire

Participants completed a brief sociodemographic form collecting general information such as age, gender, sexual orientation, marital and relational status, education level, work status, socioeconomic status, ethnicity, area of residence, and religious and political orientation.

#### Monkeypox knowledge and attitudes

A five-item *ad hoc* questionnaire based on the information published on May 23, 2022 in the report of ECDC-European Center for Disease Prevention and Control (2022b) “*Rapid risk assessment. Monkeypox multi-country outbreak*” was created to evaluate the level of knowledge about monkeypox. Questions were: “Monkeypox is caused by: […]”; “Monkeypox is transmitted: […]”; “One can be infected with monkeypox through: […]”; “The incubation period of monkeypox is: […]”; “The main symptom(s) of monkeypox is/are: […].” Five alternative answers were presented for each question, of which only one was correct. Correct answers were counted by one and summed to create the monkeypox knowledge scale.

Regarding Monkeypox attitudes, four *ad hoc* questions were assessed with response options on a six-point Likert scale (from 0—Not at all to 5—Very much). The questions were: “How much do you think monkeypox is a hoax or fake news?”; “How scared do you feel about monkeypox?”; “How much do you feel you are at risk of contracting monkeypox?”; and “How far do you think monkeypox can spread to the point of becoming a pandemic (like COVID-19)?.” Items were analyzed as separate variables and the “fake news attitude” item represented the main variable of interest for the current study.

#### COVID-19 quality of life and attitudes

An *ad hoc* questionnaire was administered with the aim of measuring the perception of one’s current quality of life (QoL; including physical health, mental health, sociality, and emotional and sexual life) and self-attributed QoL changes to the effects of the COVID-19 pandemic (see Appendix 1). An *ad hoc* questionnaire was preferred in order to capture specific self-attributions to COVID-19 rather than more general QoL measures. Higher scores indicate better general QoL (for the first variable) and greater worsening due to COVID-19 (for the second one). Exploratory factor analysis demonstrated good reliability of the measure of perceived QoL and worsening attributed to COVID-19 pandemic. In addition to these two scales, the questionnaire included single items about having contracted COVID-19 from the beginning of the pandemic, the number of vaccine doses taken, and the proximity to no-vax (anti-vaccine) positions and ideals (Zatti and Riva, 2022).

#### Epistemic trust, mistrust, and credulity questionnaire

The Epistemic trust, mistrust, and credulity questionnaire (ETMCQ) is a 15-item self-report questionnaire assessing Epistemic Trust, Mistrust, and Credulity toward communication or communicated knowledge (Campbell et al., 2021; Liotti et al., 2023). Epistemic trust refers to an adaptive attitude in relatively benign social circumstances in which the individual is selectively and appropriately open to social learning opportunities in the context of relationships. Epistemic Mistrust reflects the tendency to treat any source of information as unreliable or ill-intentioned, trying to avoid being influenced by the communication of others. Epistemic credulity refers to a marked lack of vigilance and discrimination, signaling a general lack of clarity about one’s position, and resulting in vulnerability to misinformation and the potential risk of exploitation. Higher scores indicate a higher presence of the relative trait for each factor. The validity of the ETMCQ domains demonstrated good internal consistency. The Cronbach’s alpha values for this measure in the current study ranged from 0.86 (Credulity) to 0.9 (Trust).

#### Need for closure scale

Short form of the Need for Cognitive Closure (NCS; Webster and Kruglanski, 1994; Roets and Van Hiel, 2007) which measures five dimensions related to the need for cognitive closure such as Order, Predictability, Decisiveness, Ambiguity and Close-mindedness. All these dimensions constitute separate but related aspects within the theoretical framework for the cognitive-motivational aspects of decision-making. Individuals with a high need for closure prefer order and structure in their lives, abhorring chaos, and disorder. They also prefer predictability, desire secure, and stable knowledge, and have an urgency to make quick decisions. Finally, they have a closed mindset and are unwilling to challenge their knowledge with alternative opinions or inconsistent evidence. Higher scores indicate a higher presence of the relative trait for each factor. The validity of this measure demonstrated good psychometrical characteristics. The Cronbach’s alpha values for this measure in the current study ranged from 0.78 (Decisiveness) to 0.89 (Order; Roets and Van Hiel, 2011).

#### Brief symptom inventory 18

The brief symptom inventory 18 (BSI-18) is a widely used short form of the better-known Symptom Checklist 90-Revised (SCL90-R) containing the 36-item scales related to somatization, depression, and anxiety (Derogatis, 2001). It also includes a Global Severity Index (GSI). The BSI-18 psychometric properties have already yielded satisfactory results (Frankie et al., 2017). Higher scores indicate a greater presence of symptoms reported in that domain. The Cronbach’s alpha values for this measure in the current study ranged from 0.91 (Somatization) to 0.93 (Anxiety).

#### Brief-mentalized affectivity scale

The Brief-mentalized affectivity scale (B-MAS) is a 12-item self-report questionnaire assessing three aspects of mentalized affectivity that are part of a concentric process of emotion regulation: Identifying emotions, which involves curiosity about emotions, such as naming basic emotions, and also trying to make sense of them; Processing emotions, which consists in modulating, managing, and tolerating emotions, including changing the emotion in duration or intensity; Expressing emotions, which involves the spectrum of communicating one’s feelings externally as well as internally (Greenberg et al., 2021; Liotti et al., 2021). Higher scores indicate a greater ability in mentalized affectivity. The Cronbach’s alpha values for this measure in the current study ranged from 0.88 (Identifying) to 0.91 (Processing).

#### LGBT+ and sexual attitudes

To explore attitudes toward LGBT+ people and sexuality, different short measures were assessed in this study among the one available in Italian and used in previous studies on the same population (Baiocco et al., 2014, 2020; Lingiardi et al., 2016). A visual item with drawn on a line at different distances between the respondent and the LGBT+ community was used to assess the perceived closeness between the person and the LGBT+ community. Ten items extracted from the “Attitudes toward Lesbians” and “Attitudes toward Gay Men” scales were used (Herek, 2002). Higher scores indicate more negative attitudes. The Cronbach’s alpha values for this measure in the current study ranged from 0.86 (Lesbians) to 0.89 (Gay Men). In addition, eight items extracted from the Sex-positive Attitudes Scale (Nimbi et al., unpublished) measuring sexual moralism (e.g., a woman who has sex on the first date is an easy one) was administered to investigate attitudes regarding sexual mores. Higher scores correspond to more rigid and moralistic positions toward sexuality.

#### Marlowe-crowne social desirability scale—short form

The Marlowe-crowne social desirability scale—short form (MCSDS—SF) is a 13-item measure that was developed as a means of measuring socially desirable responses (Fischer and Fick, 1993). Higher scores indicate a higher tendency to respond in a more socially desirable way. The Cronbach’s alpha value for this measure was 0.91. The MCSDS—SF was used as a covaried in the analysis of the current study to limit the effects of social desirability.

### Statistical analysis

Firstly, the primary variables of interest for the present study were presented in Table 2. Hierarchical Multiple Regression analyses (enter method; Tables 3–6) were run having “fake news attitude” as a dependent factor, and the sub-scales of each questionnaire as independent variables, to identify the significant predictors of monkeypox distrust attitude, separately for each area assessed, in line with methodological suggestions of Petrocelli (2003) and Lewis (2007): (1) demographics, socioeconomic variables, and political and religious orientation; (2), monkeypox attitudes and COVID-19 QoL and attitudes; (3) epistemic trust, need for closure, psychopathology, and mentalized affectivity; and (4) LGBT+ attitudes and sexual attitudes. A final Hierarchical Multiple Regression analysis (enter method, Table 7), including the significant variables that emerged from the previous regressions, was performed to find the best predictors of “fake news attitude.” The MCSDS—SF was used as a covaried in all the Hierarchical Multiple Regression to limit the effects of social desirability. MCSDS—SF results are presented only for the final regression for simplicity. A path analysis model was drawn with a theory-driven mode. Variables referring to more stable traits were hypnotized to have an effect in influencing behavior and attitudes variables. Fit indices used for path analysis were chi^2^ (which is not reliable for sample sizes larger than 200, but commonly used), goodness of fit (GFI), normed fit index (NFI), and comparative fit index (CFI) for which a value between 0.90 and 0.95 is considered acceptable and above 0.95 is good. Root means square error of approximation (RMSEA) is also considered, with a value of 0.01, 0.05, and 0.08 to indicate excellent, good, and mediocre fit, respectively. The width of the CI is informative about the precision in the estimate of the RMSEA (shorter interval means larger effect size). The statistical analyses were performed using IBM SPSS v. 27.0 and AMOS v.22 (SPSS Inc., Chicago, IL, United States).

**Table 2:** Monkeypox and COVID-19 variables description.

Variables		Participants (*n* = 333)
		M ± ds (min-max)
Monkeypox knowledge		3.04 ± 1.1 (0–5) Q_3_– Q_1_: 2–4
		*n* (%)
How much do you think monkeypox is a hoax or fake news? (fake news attitude)	Not at all	206 (61.86)
	Little	75 (22.52)
	Moderately	23 (6.91)
	Quite	10 (3)
	Very	7 (2.1)
	Very much	12 (3.6)
Where did you mainly acquire your knowledge about monkeypox?	From the news and newspapers	109 (32.73)
	From social media	80 (24.02)
	From friends, relatives, and colleagues	18 (5.41)
	I have searched info on internet	86 (25.83)
	Books and other scientific fonts	20 (6.01)
	I have not received any information about monkeypox	20 (6.01)
How scared do you feel about monkeypox?	Not at all	82 (24.62)
	Little	141 (42.34)
	Moderately	75 (22.52)
	Quite	25 (7.51)
	Very	6 (1.8)
	Very much	4 (1.2)
How much do you feel at risk of contracting monkeypox?	Not at all	92 (27.63)
	Little	164 (49.25)
	Moderately	61 (18.31)
	Quite	9 (2.7)
	Very	3 (0.9)
	Very much	4 (1.2)
How far do you think monkeypox could spread so far as to become a pandemic (as happened with COVID-19)?	Not at all	70 (21.02)
	Little	151 (45.35)
	Moderately	80 (24.02)
	Quite	20 (6.01)
	Very	7 (2.1)
	Very much	5 (1.5)
Number of times COVID-19 has been contracted	Never	151 (45.34)
	Once	158 (47.45)
	Twice	24 (7.21)
Number of doses of antiCOVID-19 vaccine administered	Zero	34 (10.21)
	One	2 (0.6)
	Two	46 (13.81)
	Three	247 (74.17)
	Four	4 (1.2)
How much do you feel in agreement with the NO-VAX positions expressed during the COVID-19 pandemic?	Totally disagree	189 (56.76)
	Partially disagree	30 (9.01)
	Neither in agreement nor disagreement	25 (7.51)
	Partially agree	36 (10.81)
	Totally agree	53 (15.92)

**Table 3:** Hierarchical multiple regression analyses on sociodemographic variables (*n* = 333).

	*B*	*SE*	*β*
*1.1 Demographics such as age, gender, sexual orientation, and relationship status*			
Age	0.023	0.007	0.206^***^
Gender (Female = 0/Male = 1)	0.042	0.165	0.016
Sexual orientation (Heterosexual = 0/LGB+ = 1)	−0.495	0.164	−0.177^**^
Being in a relationship (No = 0/Yes = 1)	0.059	0.122	0.027
*1.2 Socioeconomic variables*			
Education level	−0.017	0.089	−0.011
Socio-economic status	0.078	0.082	0.054
Residence area (From the metropolis to the rural area)	0.021	0.057	0.020
*1.3 Political and religious orientation*			
Political conservativisms (Right winged)	0.229	0.069	0.205^***^
Religious education	0.233	0.067	0.211^***^
Religiousness	0.231	0.064	0.227^***^

^*^p < 0.05; ^**^p < 0.01; ^***^p < 0.001.

Social desirability was put as a covariate in every regression. Results are not reported for simplicity.

**Table 4:** Hierarchical multiple regression analyses on Monkeypox and COVID-19 related variables (*n* = 333).

	*B*	*SE*	*β*
*2.1 Monkeypox attitudes*			
Monkeypox knowledge	−0.346	0.056	−0.320***
Monkeypox fear	−0.205	0.078	−0.174**
Monkeypox perceived risk of contagion	0.097	0.086	0.073
Monkeypox spread as a pandemic	0.006	0.072	0.005
*2.2 COVID-19 QoL and attitudes*			
Current perceived QoL	−0.013	0.017	−0.041
Worsening QoL attributed to the COVID-19 pandemic	0.019	0.014	0.068
Had COVID-19 (No = 0/Yes = 1)	−0.226	0.103	−0.108*
Number of COVID-19 vaccine doses made	−0.591	0.071	−0.453***
Agreement with no-vax positions	0.173	0.043	0.214***

^*^p < 0.05; ^**^p < 0.01; ^***^p < 0.001.

QoL, Quality of Life.

Social Desirability was put as covariate in every regression. Results are not reported for simplicity.

**Table 5:** Hierarchical multiple regression analyses on psychological variables (*n* = 333).

	*B*	*SE*	*β*
*3.1 Epistemic trust*			
Trust	−0.053	0.013	−0.244^***^
Mistrust	0.025	0.011	0.136^*^
Credulity	0.026	0.020	0.081
*3.2 Need for closure*			
Order	−0.130	0.062	−0.146^*^
Predictability	0.035	0.077	0.036
Decisiveness	−0.140	0.073	−0.138
Ambiguity	−0.036	0.087	−0.034
Close-mindedness	0.178	0.080	0.152^*^
*3.3 Psychopathology*			
Somatization	−0.155	0.133	−0.085
Depression	−0.067	0.110	−0.045
Anxiety	0.210	0.120	0.142
*3.4 Mentalized affectivity*			
Identifying	−0.018	0.014	−0.075
Processing	0.032	0.014	0.133^*^
Expressing	−0.017	0.012	−0.086

^*^p < 0.05; ^**^p < 0.01; ^***^p < 0.001.

Social Desirability was put as covariate in every regression. Results are not reported for simplicity.

**Table 6:** Hierarchical multiple regression analyses on LGBT+ and sexual attitudes (*n* = 333).

	*B*	*SE*	*β*
*4.1 LGBT+ attitudes*			
Closeness to LGBT+ community	0.023	0.036	0.033
Negative attitudes toward gay men	0.125	0.038	0.365^***^
Negative attitudes toward lesbians	0.015	0.040	0.041
*4.2 Sexual attitudes*			
Moralism	0.063	0.007	0.455^***^

^*^p < 0.05; ^**^p < 0.01; ^***^p < 0.001.

Social Desirability was put as covariate in every regression. Results are not reported for simplicity.

**Table 7:** Final hierarchical multiple regression analyses on best predictors emerged (*n* = 333).

	*B*	*SE*	*β*
*Final regression—Best predictors*			
Step 1			
*Social desirability*	−0.052	0.032	−0.110
Step 2			
*Social desirability (covariate)*	−0.020	0.028	−0.041
Age	−0.006	0.007	−0.060
Sexual orientation (Heterosexual = 0/LGB+ = 1)	−0.097	0.140	−0.039
Political conservativisms (Right winged)	0.018	0.078	0.015
Religious education	0.078	0.066	0.068
Religiousness	0.146	0.069	0.139*
Monkeypox knowledge	−0.194	0.056	−0.191***
Monkeypox fear	−0.064	0.068	−0.054
Had COVID-19 (No = 0/Yes = 1)	−0.176	0.103	−0.096
Number of COVID-19 vaccine doses made	−0.302	0.089	−0.225***
Agreement with no-vax positions	0.120	0.044	0.163**
Trust	−0.025	0.012	−0.129*
Mistrust	0.028	0.011	0.153**
Order	−0.059	0.052	−0.070
Close-mindedness	0.031	0.071	0.026
Processing	−0.001	0.014	−0.004
Negative attitudes toward gay men	0.058	0.026	0.178*
Moralism	0.013	0.013	0.092

^*^p < 0.05; ^**^p < 0.01; ^***^p < 0.001.

## Results

The sociodemographic characteristics of the group are presented in Table 1. The mean age of participants was 31.71 (ranging between 18 and 71), with most respondents under 40 years old (Q1). Two-thirds of the group were females assigned at birth, self-identifying as female. Most participants report being heterosexual, unmarried, and in a relationship (about 3% are polyamorous). Concerning socioeconomic status, the group has medium/medium-high schooling, and half of the participants report being employed. 97% of the group reports being white Caucasian, living in a large city (53.82%), and having medium/medium-low socioeconomic status. For political and religious orientation, the group comprises 45.9% of people who identify with the center-left (moderate progressives), 52.85% report professing no religion, and 39% report being Christian Catholic. Regardless, 94% report having had a religious upbringing.

Some descriptive data about attitudes and knowledge toward monkeypox and COVID-19 are presented in Table 2. Regarding monkeypox knowledge, the group seems balanced between those who correctly answered the questions and those who did not. Most of the group seems to have informed themselves through newspapers and TV news, followed by those who sought information on the Internet and social media. With respect to attitudes toward monkeypox, more than 60% did not believe that monkeypox is fake news. Most of the group seemed to report that they were a little frightened of this pathogen, felt little risk of contracting the virus, and had little fear that monkeypox could become a pandemic, as happened with COVID-19. Regarding adherence to health policies regarding COVID-19, more than half of the group said they had contracted COVID at least once, and about 75% have received three doses of the anti-COVID vaccine versus about 10% who have not received a single dose. Almost a quarter of the group seems to agree, at least in part, with no-vax (anti-vaccine) positions expressed during the COVID-19 pandemic.

Regarding the sample size and the statistical power level, a minimum of 149 participants was calculated *a-priori* as large enough to run the following analyses, having a desired statistical power 0.8, 18 predictors, a minimum effect size of 0.15 (considered medium), and a probability level set at 0.05. The actual sample size for the Hierarchical Multiple Regression analyses is composed by 333 participants. The *post-hoc* statistical power, with observed *R*^2^ of 0.45, 18 variables, and a probability level set at 0.05, reported an observed statistical power of 0.99.

To assess the best predictors of fake news attitudes toward monkeypox, several classes of Multiple Hierarchical Regressions analyses (enter method, Tables 3–6) were performed, having the social desirability as covariate and the domains of each questionnaire as independent variables. The predictors age, sexual orientation, political conservatism, religious education, religiousness, Monkeypox knowledge and fear, having had COVID-19, the number of COVID-19 vaccine doses administrated, agreement with no-vax positions, epistemic trust, and mistrust, order and close-mindedness traits, processing emotions, negative attitudes toward Gay men, and sexual moralism significantly emerged from the respective regression models. Specifically, higher levels of monkeypox fake news attitudes were associated to older participants, being heterosexual, politically conservative, having had a religious education and giving more importance to religion, having more negative attitudes toward gay men, more sexual moralism, having less knowledge and fear about monkeypox, not having contracted COVID-19, having had administered a lower number of COVID-19 vaccine doses and being closer to no-vax ideas. On the psychological side, higher levels of monkeypox fake news attitudes were associated with lower levels of epistemic trust and order traits, Conversely, higher levels of monkeypox fake news attitudes were associated with higher levels of epistemic mistrust, close-mindedness, and ability to process emotions.

To highlight the best predictor of fake news attitudes, a final Hierarchical Multiple Regression was conducted having social desirability as covariate (Table 7, Step 1), and using the factors significantly emerged in the previous analyses as predictors (Table 7, Step 2). The analysis revealed a significant general model explaining the 45.2% of variance in monkeypox fake news attitudes [*F*_(18,315)_ = 9.21, *p* < 0.001, Δ*R*^2^ = 0.45]. The predictors religiousness, monkeypox knowledge, number of COVID-19 vaccine doses administrated, agreement with no-vax positions, trust, mistrust, anxiety, and negative attitudes toward gay men emerged as significant predictors of monkeypox fake news attitude. In contrast, monkeypox knowledge appeared to be the strongest one.

**Figure 1:**
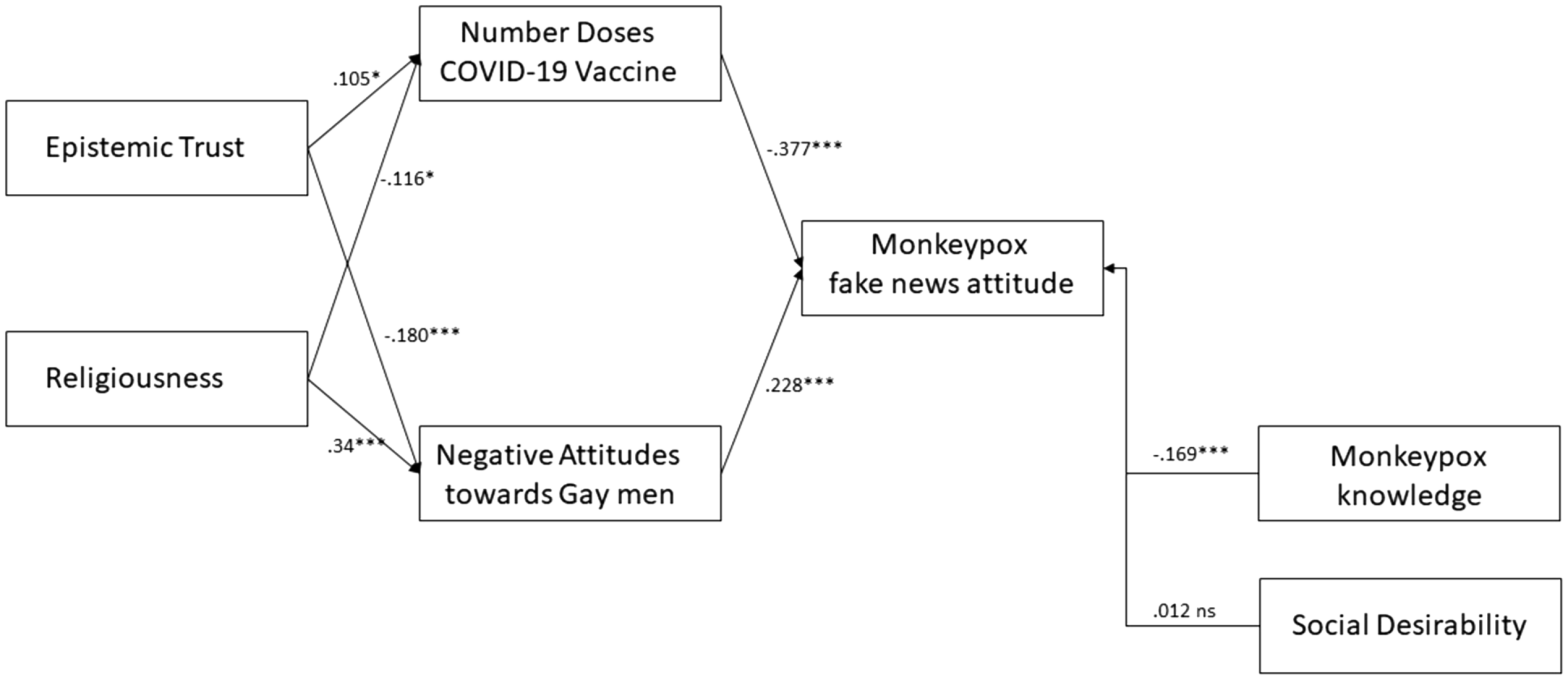
Path diagram full mediation model of Epistemic Trust and Religiousness on Monkeypox fake news attitude.

After excluding two variables (“epistemic mistrust” and “agreement with no vax positions”), whose theoretical proximity and collinearity posed risks of model misinterpretation, the path diagram presented in Figure 1 was constructed. The variables were organized with paths that consider not only their direct impact on attitudes toward monkeypox as fake news but also the interaction between them. The main variables selected were epistemic trust and religiosity, while the number of vaccine doses and negative attitudes toward gay men were identified as endogenous variables, dependent on trust and religiosity. Monkeypox knowledge and social desirability (covariate) were positioned as exogenous variables, considering their independence from the other predictors.

Considering a sample size of 333 subjects that could lower the power of chi^2^ based analyses, the model was tested showing a satisfactory fit to data (chi^2^ = 19.142, df = 12, *p* = 0.085; GFI = 0.962; NFI = 0.904; CFI = 0.96; RMSEA = 0.042 [95% CI.000–0.076]). As shown in Figure 1, all the endogenous paths were found to be significant. Monkeypox knowledge showed a significant effect on fake news attitude. The standardized total effects of Trust and Religiousness on monkeypox fake news attitude were medium (Trust = −0.081, *p* = 0.009; Religiousness = 0.121, *p* < 0.001). Regarding mediations, trust and religiousness were significantly indirectly connected with fake news attitudes through the number of vaccine doses made and negative attitudes toward gay men. This model explained the 25% of variance in monkeypox fake news attitude.

## Discussion

In line with the study’s objective to explore some characteristics related to monkeypox fake news attitudes in the early 2022 western outbreak, an attempt was made to reach a varied group of Italian participants pertaining to sociodemographic characteristics. Regarding information and knowledge about monkeypox based on the report of ECDC-European Center for Disease Prevention and Control (2022b), an acceptable variation emerged among participants, suggesting how the Italian population has differently acknowledged information in this delicate phase (June 2022). Most participants showed limited fear or anxiety regarding monkeypox, feeling not at risk of infection and reporting low levels of worry about the possibility that it could become a pandemic such as COVID-19. At the time of closing data collection (1st July 2022), 192 cases of monkeypox were confirmed in Italy (Italian Ministry of Health, 2022), with a primary prevalence of men (*n* = 190) and 35.42% cases related to travel abroad. The report does not mention the sexual orientation of the men infected or the countries where they have traveled. However, the male prevalence and the connection to travel may influence the perceived dangerousness for the general population as something very distant from their everyday life and limited to MSM coming from LGBT+ international events (Mileto et al., 2022), as it was initially for HIV more than 40 years ago (Hedge et al., 2021).

Following the first aim of the current study, some interesting variables connected to the monkeypox fake news attitudes emerged: participants who were more likely to interpret monkeypox news as a hoax were significantly more likely to be older, heterosexual, politically conservative (right-winged), with a higher level of religious education, and reporting higher importance given to religion in their lives. A study on COVID-19 showed that older people were more susceptible to being influenced by fake news as well as spreading inaccurate information (Sun et al., 2020). It could therefore be theorized that older age and less familiarity with social media may devalue this source of information for monkeypox as well. This element needs to be confirmed in future studies since it might stress the need for a different and more accurate type of health communication for older people. The heterosexual orientation as a predictor of fake news attitudes could be explained by the fact that LGBT+ people (especially MSM) seem more at risk of monkeypox in this phase, in line with the early reports (Bragazzi et al., 2022; Schmidt-Sane et al., 2022). As said, heterosexual people might feel the risk of monkeypox as distant and, in that sense, more likely to regard it as an unfounded alarm or fake news. At the same time, having more negative attitudes toward gay men and higher levels of sexual moralism (view of sexuality as ethically acceptable only if for reproductive purposes within a married couple) seems to be connected to monkeypox as fake news. These elements are central to health policies because viewing an infectious disease as something that does not affect the general population but affects only a sexual minority or “unmoral behaviors,” lowers the attention and increases the risk of infection. In that sense, Schmidt-Sane et al. (2022) emphasize the need to broaden health communication by referring to transmission routes rather than sexual orientation, avoiding misconceptions that can trigger the activation of different levels of stigma from an intersectional perspective (Nimbi et al., 2021; Schmidt-Sane et al., 2022).

The discourse on political orientation and religiosity is complex and may be connected to attitudes toward sexuality. The relationship between media, fake news, and political ideology has been widely investigated, especially in the English-speaking context. Many studies (e.g., Axt et al., 2020; Calvillo et al., 2020; Steinfeld, 2021; Wisker and McKie, 2021) have identified how conservative political orientations are more likely to be associated with a greater tendency to disbelieve information (especially those from authoritative media), having a higher need for cognitive structure and belief in intentional deception of news, that is, the need to simplify and having information that is unbiased and unambiguous. For example, in the study of Calvillo et al. (2020) on COVID-19, conservatism was associated with perceiving less vulnerability to the infection, and stronger endorsement of the beliefs that the spread of the virus was a conspiracy and media had exaggerated its impact. Moreover, conservatism predicted less accurate selectivity between real and fake news. Much of what has been discussed regarding political orientation also applies to religiousness (Wisker and McKie, 2021). In the current study, most participants who received a religious education or declared to confess a religion were Catholic Christians. Stefanone et al. (2019) highlighted religiosity as a positive predictor of fake news’ credibility in general and a higher incidence of perceived fake news, showing religious people being more prone to believe COVID-19-related misleading and conspiracy narratives (Buturoiu et al., 2021). In this sense, how religion responds to societal inputs is essential, since it seems to be able to influence attitudes and health behaviors (Barringer and Savage, 2022). Moreover, some studies on HIV showed that when pathology is associated with sexual behavior and minorities, stigma is strongly associated with religious beliefs such as “HIV is a punishment from God” or that “people living with HIV/AIDS are sinner,” with possible disruptive effects on physical and mental health (Zou et al., 2009; Medved Kendrick, 2017). Therefore, how politics and religion can foster trust in health information should be taken seriouslyin health information should be taken seriously, involving political and religious leaders, who play a central role in conveying reliable content to the audience in managing effective healthcare strategies.

Other important suggestions may come from analyzing results related to monkeypox knowledge and specific attitudes toward COVID-19. Greater knowledge of monkeypox and fear are associated with less tendency to consider monkeypox a hoax. This element underscores the importance of conveying information that is scientifically correct and possibly clear and simple (Schmidt-Sane et al., 2022), investing in the process of health education, which includes the development of critical thinking and through a comprehensive sexual education (Todaro et al., 2018; Nimbi et al., 2021). A lower level of education could affect less specific knowledge and less agency in addressing a health risk (Di Crosta et al., 2020).

Regarding participants’ experience with COVID-19 pandemic, the perception of monkeypox as fake news seems to be associated with never having had COVID-19, having fewer doses of vaccine administered, and agreeing with no-vax positions. These results would seem to underscore the presence of a broader dimension related to adherence to conspiracy and denialist theories that have been widely held during the COVID-19 pandemic (Brainard and Hunter, 2020; Das and Ahmed, 2022) that might be extended to the spread of other pathogens such as monkeypox and related health strategies (e.g., vaccination). The role of these ideologies has been much discussed in the recent literature, noting a significant threat to health risk management and prevention policies. Some authors (Brainard and Hunter, 2020; Moscadelli et al., 2020; Pulido et al., 2020; Gupta et al., 2022) have suggested possible strategies to mitigate their effects on public health as contrasting the circulation of harmful information and educating people to discriminate between good and bad advice in diseases outbreaks. Messages focusing on fake health information are often aggressive, while those based on scientific evidence are usually expressed respectfully and applicatively (Pulido et al., 2020).

Regarding the reliability of the information, epistemic trust and mistrust are psychological constructs that may play a significant role. In the current study, they emerged among the main predictors of monkeypox fake news attitudes. As the authors of the ETMCQ theorized (Campbell et al., 2021), epistemic trust refers to an adaptive stance in which the individual is selectively and appropriately open to opportunities for social learning, a factor that may confer psychological resilience in life challenges. In contrast, epistemic mistrust indicates the tendency to treat any source of information coming from others as unreliable or malevolent. So these traits may partly explain people’s general tendency to believe and perceive information from the environment as useful, make it their own, or reject it, regardless of the content.

Lower scores on order and being more close-minded emerged as associated with higher levels of fake news attitudes. Close-mindedness describes the desire for secure closure, the unwillingness to have one’s knowledge challenged and made unsafe by alternative opinions or inconsistent evidence (Webster and Kruglanski, 1994). One possible explanation is that having a major cognitive need for closure makes straightforward the reaction to a potential threat as the monkeypox epidemic, activating stereotypical and stigmatizing thoughts, such as declassing the threat as a hoax. This cognitive mechanism may help to reestablish order avoiding the feeling of danger. A study on COVID-19 (Dagli, 2020) suggested that when the possible causes of the epidemic spread are not clear and specific, close-minded attitudes, and rumors often increase, leading to fear, stigma, and negative behaviors as the dramatic cases of violence and suicides in the first pandemic outbreak. Connected with the idea of the need for closure and mistrust, higher scores on processing emotions resulted in associated attitudes toward monkeypox as fake news. It seems that the more people can manage their emotions and keep them under control, the more they will try to ward off possible emotional threats to their internal balance, defining these potential dangers as fake news. The fear of an invisible enemy one has just heard about (monkeypox) in a context dominated by fear and fatigue in dealing with a pandemic (COVID-19) could make emotional processing very complex, also resulting in emotional discharge and denial (Jurist, 2018).

In line with the second objective of this study, results about epistemic trust and mistrust should be discussed together with monkeypox and COVID-19 attitudes and religiousness. In particular, the close relationship between monkeypox knowledge, the number of COVID-19 vaccine doses, and proximity to no-vax positions might make us think about how central the role of trust/mistrust is in the current sociocultural and historical context, characterized by a large flow of information, often contradictory, that can undermine the sense of confidence toward science and health policies (Hendriks et al., 2016). Recognizing the role of individual traits and further exploring novel constructs such as epistemic trust and mentalized affectivity (Jurist, 2018; Greenberg et al., 2021; Tanzilli et al., 2022) might be essential for designing more effective programs in public health. Raising awareness and reducing epistemic mistrust may be crucial to enable improved adaptation to interpersonal environments, increasing the effectiveness of health strategies ranging from prevention campaigns to psychotherapeutic interventions. This argument applies perfectly to the current monkeypox outbreak in Italy and other western countries, but it can also be easily extended to broad health communication. A general recommendation is to consider what we have learned from past epidemics like HIV/AIDS and COVID-19. In both cases, we have assisted to the dissemination of information that has fueled confusion, dissatisfaction, fear, and anger and ended in discrimination toward specific groups (MSM, transgender people, sex workers, people who inject drugs, and Chinese people), as well as preventing the fight against the pathology (Starks et al., 2013; Hedge et al., 2021; Schmidt-Sane et al., 2022). In this sense, the mediation model presented represents an attempt to describe how variables describing structural aspects such as traits (epistemic trust and religiosity) can have an indirect effect on the interpretation of health information related to attitudes toward sexuality (negative attitude toward gay men) and the practice of health-related behavior linked to a previous health emergency (number of COVID-19 vaccine doses carried out).

The present study has some limitations that should be recognized. (i) Participants were recruited with a “snowball” technique and advertisement on social media; therefore, it is not possible to generalize the results for the Italian population despite the good variability of participants involved. (ii) Some measures were *ad hoc* developed to measure reactions and attitudes toward monkeypox and COVID-19. Although preliminary psychometric analyses have been conducted, further studies should investigate the validity and reliability of these constructs. (iii) This study was composed of self-report questionnaires. In this sense, the responses can be easily falsified by respondents. To limit this bias, the social desirability measure was considered and used as a control variable.

Future studies should consider extending the investigation of monkeypox reactions to explore the differences and similarities between countries. Another issue is to develop a survey within the LGBT+ community (especially among MSM), to assess the evolution of reactions and associated distress (Bragazzi et al., 2022).

## Conclusion

An evaluation of psychological and social predictors of monkeypox fake news attitudes in Italy has been presented in the current study. Some significant factors included epistemic trust and mistrust, monkeypox knowledge, number of COVID-19 vaccine doses administered, agreement with no-vax positions, religiousness, and negative attitudes toward gay men. These factors, together with the others that emerged from the study, may be central to directing health strategies in the prevention and psychological support field. This data can help to inform practitioners and policymakers in their efforts to manage the western monkeypox outbreak. In addition, specific profiles of people who may be more at risk of believing monkeypox to be a hoax or conspiracy (e.g., older age, heterosexual sexual orientation, political conservativism, and religiousness) have been identified. Knowing these characteristics may be useful in improving the effectiveness of health communication, designing targeted education, and supporting such individuals to engage in healthier behaviors.

Although there is significant uncertainty about current monkeypox outbreaks, resources based on experience from past infectious disease outbreaks must be considered (Schmidt-Sane et al., 2022). This is a critical time to put these lessons into practice and ensure that risk communication is evidence-based and is taken seriously, expanding consideration of those at risk and focusing on collaborative communities and institutions.

## Data availability statement

The raw data supporting the conclusions of this article will be made available by the authors, without undue reservation.

## Ethics statement

The studies involving human participants were reviewed and approved by Ethical committee of the Department of Dynamic and Clinical Psychology and Health Studies, Sapienza University of Rome, Italy. The patients/participants provided their written informed consent to participate in this study.

## Author contributions

FN, RB, and VL originated the idea and design for the current study. FN, GG, and AT conducted literature searches, provided summaries of previous research studies, followed the investigation process, and performed the data collection. FN wrote the first draft of the manuscript. RB, GG, AT, and VL supervised the research process and the final draft of the manuscript. All authors contributed to the article and approved the submitted version.

## Conflict of interest

The authors declare that the research was conducted in the absence of any commercial or financial relationships that could be construed as a potential conflict of interest.

## Publisher’s note

All claims expressed in this article are solely those of the authors and do not necessarily represent those of their affiliated organizations, or those of the publisher, the editors and the reviewers. Any product that may be evaluated in this article, or claim that may be made by its manufacturer, is not guaranteed or endorsed by the publisher.

## Appendix

Appendix 1 *Ad hoc* quality of life COVID-19 related measure used for the study with factor loadings.

**Table:**

Items	Current perceived QoL	Worsening QoL attributed to the COVID-19 pandemic
1.How do you evaluate your current physical health?	**0.569**	0.010
2.How do you evaluate your current state of psychological well-being?	**0.676**	−0.164
3.How do you evaluate your current social life?	**0.759**	−0.127
4.How do you evaluate your current emotional life?	**0.835**	−0.113
5.How do you evaluate your current sex life?	**0.770**	−0.121
6.How much do you think the COVID-19 pandemic and its social effects (such as lockdowns and other restrictions) have NEGATIVELY INFLUENCED your current physical health?	−0.022	**0.691**
7.How much do you think the COVID-19 pandemic and its social effects (such as lockdowns and other restrictions) have NEGATIVELY INFLUENCED your current state of psychological well-being?	0.022	**0.765**
8.How much do you think the COVID-19 pandemic and its social effects (such as lockdowns and other restrictions) have NEGATIVELY INFLUENCED your current social life?	−0.087	**0.815**
9.How much do you think the COVID-19 pandemic and its social effects (such as lockdowns and other restrictions) have NEGATIVELY INFLUENCED your current emotional life?	−0.253	**0.805**
10.How much do you think the COVID-19 pandemic and its social effects (such as lockdowns and other restrictions) have NEGATIVELY INFLUENCED your current sex life?	−0.257	**0.698**

Principal component Analisis—Bartlett’s test of sphericity: Chi^2^ = 1331.738, *p* < 0.001; Total variance explained = 57.179%.
